# Model Resuscitation Leadership Curriculum for Emergency Medicine Residents: Modified Delphi Study

**DOI:** 10.5811/westjem.50811

**Published:** 2026-03-01

**Authors:** Michael Sobin, Peter Prescott, David Berger, Danielle Turner-Lawrence, Brett Todd

**Affiliations:** * Medical College of Wisconsin, Department of Emergency Medicine, Milwaukee, Wisconsin; † Oakland University William Beaumont School of Medicine, Rochester, Michigan; ‡ Corewell Health William Beaumont University Hospital, Department of Emergency Medicine, Royal Oak, Michigan

## Abstract

**Objectives:**

Effective resuscitation leadership is essential for emergency physicians, yet formal training in this domain remains limited within emergency medicine (EM) residency programs. Generic healthcare teamwork frameworks do not fully address the unique demands of EM resuscitations, including diagnostic uncertainty, time pressure, and frequent interruptions. Without consensus on the key competencies or instructional strategies needed to teach these EM-specific resuscitation leadership skills, residency programs lack clear curricular guidance. We aimed to achieve expert consensus on the learning objectives and educational strategies for a longitudinal model resuscitation leadership curriculum for EM residents using a modified Delphi approach.

**Methods:**

We conducted a three-round modified Delphi study from September 2024–March 2025. Panelists were selected based on expertise in resuscitation leadership education and scholarship. We conducted a PubMed literature review that identified 19 references encompassing 244 skills and synthesized them into 31 initial learning objectives. By consensus, 12 educational strategies were identified. Panelists rated the importance of proposed learning objectives and educational strategies derived from a review of the literature and existing assessments. Additional items were added and refined across rounds based on panelist feedback. Consensus thresholds were predefined as > 75% agreement for inclusion (rated as important/very important or agree/strongly agree).

**Results:**

Twelve experts participated in the study, representing diverse institutions and training backgrounds. By Round 3, consensus was achieved for 28 learning objectives and 13 educational strategies. Items were thematically categorized, and supplemental resources were developed to guide curricular implementation. The final curriculum integrates cognitive, procedural, and non-technical competencies contextualized within resuscitation environments and sequenced to support longitudinal skill development.

**Conclusion:**

This study presents the first expert consensus-derived resuscitation leadership curriculum for EM residents. The resulting framework provides EM residency programs with adaptable, evidence-informed guidance to support structured, longitudinal resuscitation leadership training and improved resuscitation team performance.

## INTRODUCTION

Effective resuscitation leadership is a critical competency for emergency physicians, given the frequency and complexity of medical resuscitations encountered in the emergency department (ED).^1^ From the outset of residency, emergency medicine (EM) programs expect trainees not only to apply clinical knowledge and perform life-saving procedures but also to lead interdisciplinary teams under pressure, communicate clearly, and make rapid decisions.^2^ These responsibilities demand a diverse skillset, including technical expertise, cognitive agility, and interpersonal competence.^3^ Moreover, leadership during resuscitation has been shown to significantly influence both team performance and patient outcomes.^4^–^6^

Despite widespread recognition of its importance, formal training in resuscitation leadership remains limited within EM residency programs. Graduate medical education organizations in EM provide minimal guidance regarding the specific components of resuscitation leadership that educators should teach, with only a few published curricula addressing this need.^1^,^7^,^8^ Although other medical specialties and labor sectors have developed structured approaches to resuscitation and team leadership training, including Crisis Resource Management (CRM) and Team Strategies and Tools to Enhance Performance & Patient Safety (TeamSTEPPS), the applicability of these models to the unique context of EM resuscitations remains unclear.^9^–^11^

Resuscitations in the ED differ substantially from those in operating rooms, intensive care units, and inpatient rapid-response environments, where patient information is often more complete, staffing ratios more favorable, and interruptions less frequent. In contrast, resuscitations in the ED occur in an environment characterized by undifferentiated patients, higher cognitive load arising from constant interruptions, boarding pressures, ad hoc team composition, and escalating administrative demands.^12^ These factors impair the consistent execution of leadership behaviors linked to improved resuscitation performance. For example, stronger leadership performance has been associated with shorter time to defibrillation, reduced hands-off time, faster intubation, and more appropriate allocation of procedural tasks.^4^,^5^ Moreover, generic healthcare team-training such as CRM and TeamSTEPPS may overlook the realities of ad hoc EM teams, which cannot rely on pre-established shared mental models and, thus, require context-specific strategies to rapidly establish team understanding.^13^ Therefore, while CRM and TeamSTEPPS offer valuable foundational principles, neither approach fully addresses the specialized knowledge and skills needed for competent resuscitation leadership in EM. This persistent gap underscores the need for targeted, EM-specific resuscitation leadership training for EM residents.

In practice, residents often report receiving resuscitation and team leadership instruction informally, through observation or the “hidden curriculum” within clinical environments.^1^,^14^ This apprenticeship model, while longstanding, may be insufficient in training settings where opportunities for observation, coaching, and debriefing are non-standardized and time limited.^8^,^15^ Resuscitation certifications (Advanced Cardiovascular Life Support, Advanced Trauma Life Support, Basic Life Support, and Pediatric Advanced Life Support) focus primarily on algorithmic clinical management and offer limited instruction in the non-technical leadership competencies essential to effective team performance.^4^ Likewise, the Accreditation Council for Graduate Medical Education (ACGME) EM Milestones provide a general framework for competency-based progression but lack specificity regarding leadership during resuscitative care.^16^

**Population Health Research Capsule**

What do we already know about this issue?

*Effective resuscitation leadership improves patient outcomes, yet which competencies and learning methods prepare EM residents to lead resuscitation is unclear*.

What was the research question?

*What learning objectives and educational methods should guide a resuscitation leadership curriculum for EM residents?*

What was the major finding of the study?

*Using a modified Delphi process, 12 experts reached consensus on 28 learning objectives and 13 educational strategies*.

How does this improve population health?

*Incorporating this model resuscitation leadership curriculum may improve leader performance, team dynamics, and care for critically ill patients in the emergency department*.

To date, no consensus-driven effort has defined the knowledge, skills, and behaviors that constitute effective resuscitation leadership for EM residents. While some resuscitation leadership curricula have been developed, they vary in scope and lack a unified framework for integration into longitudinal residency training.^7^,^8^ Given this gap, the next step in curriculum development, as per Kern’s six-step approach, is to define specific learning objectives and determine the ideal instructional strategies for teaching resuscitation leadership.^17^ This widely used curriculum-development methodology provides a structured, evidence-based framework grounded in the needs of learners, educators, and healthcare systems.

Our objective in this study was to achieve expert consensus on the learning objectives and educational strategies for a model longitudinal resuscitation leadership curriculum for EM residents using a modified Delphi approach.

## METHODS

### Study Design

We used a modified Delphi methodology to achieve consensus on the learning objectives and educational methods for a model resuscitation leadership curriculum for EM residents.^18^ The Delphi technique is a consensus-building methodology that has been previously applied to develop curricular components in EM graduate medical education and has extensive validity evidence.^19^–^22^ Consistent with best practices for Delphi studies, our approach included systematic identification of the problem area, defined criteria for expert panelist selection, iterative discussion with controlled feedback during Delphi rounds, strict anonymity of panel members, and a priori determination of consensus and stopping criteria.^18^ The institutional review board at Corewell Health William Beaumont University Hospital approved this study under exempt status.

### Settings and Participants

We aimed to recruit 15 to 20 resuscitation leadership experts from a diverse range of institutions to serve as expert panelists, a target consistent with previous modified Delphi studies on curriculum development.^19^–^22^ Potential panelists were identified using predefined inclusion criteria: 1) at least one peer-reviewed publications related to resuscitation, resuscitation leadership, or resuscitation education; 2) completion of critical care or resuscitation fellowship training; or 3) documented involvement in developing resuscitation educational products, such as curricula, seminars, or coaching programs. We also prioritized a broad variety in practice settings and geographic regions. Panel candidates were identified through a combination of author knowledge of recognized resuscitation leaders, PubMed searches informed by the preliminary review of the literature focused on knowledge and skills, and targeted online searches related to EM resuscitation leadership. After identifying eligible candidates using predefined criteria, we recruited panelists via direct email. All survey distributions and communications with the expert panel were conducted through REDCap, hosted at William Beaumont University Hospital.^23^,^24^ The Delphi process was carried out from September 2024–March 2025.

### Study Protocol

We developed a definition of resuscitation leadership in EM to ensure clarity and consistency during curriculum development. First, the study team internally defined a resuscitation as “the evaluation and management of life-threatening medical emergencies requiring immediate stabilization.” Next, we conducted a review of the available literature to inform the definition of resuscitation leadership using a PubMed search with the keywords “resuscitation” and “leadership.” Additionally, to inform the definition we examined the leadership conceptual model proposed by Kozlowski et al and applied to EM teams by Rosenman, Branzetti, and Fernandez to inform the definition.^16^,^25^ Resuscitation leadership was defined as follows: “The act of guiding a team during life-threatening medical emergencies to optimize patient outcomes.” To ensure validity, this definition was reviewed by two institutional resuscitation experts.

We then identified the initial learning objectives for expert panel review by determining the relevant knowledge and skills expected of a competent resuscitation leader. Given the limited number of published resuscitation leadership curricula, we expanded the search to include available resuscitation leadership assessments. After conducting a PubMed search using the keywords “resuscitation leadership” and “resuscitation leadership assessment,” we included articles that explicitly listed distinct resuscitation leadership knowledge and skills for a specific curriculum, educational offering, or assessment. Additionally, we reviewed bibliographies to identify additional studies meeting the inclusion criteria. This process identified 19 curricula, educational offerings, and assessments, yielding a total of 244 resuscitation leadership knowledge items and skills.^4^,^6^,^9^,^10^,^26^–^39^ We categorized these into similar groups, resulting in an initial list of 31 learning objectives for expert panel consideration. Additionally, we identified 12 initial educational strategies based on internal consensus.

In each Delphi round, expert panelists completed a digital survey that ensured strict anonymity of panelists and their responses. For each round, panelists rated learning objectives on a five-point Likert scale based on its importance in ensuring EM residents become competent resuscitation leaders (1 = Not important, 2 = Slightly important, 3 = Moderately important, 4 = Important, 5 = Very important). Similarly, panelists evaluated educational strategies using a five-point Likert scale to determine its suitability for teaching resuscitation leadership to EM residents (1 = Strongly disagree, 2 = Disagree, 3 = Neither agree nor disagree, 4 = Agree, 5 = Strongly agree). Panelists were also invited to provide narrative comments, suggesting modifications or additions to the proposed learning objectives and educational strategies. We piloted the initial survey with a faculty member at our institution and one of our residency graduates with expertise in medical education research. The wording of the learning objectives, educational strategies-survey questions, and associated Likert-scale were refined based on pilot feedback.

We designed the study to conclude after three Delphi rounds, with consensus cutoffs determined a priori. Positive consensus was defined as greater than 75% agreement on items rated as very important/strongly agree or important/agree. Negative consensus was defined as greater than 75% agreement on items rated as not important/strongly disagree or slightly important/disagree. These thresholds align with established Delphi methodology for curriculum development.^22^ We included items meeting positive consensus in the final curriculum’s learning objectives and educational strategies. Conversely, items meeting negative consensus or failing to reach consensus after the predetermined three rounds were excluded from consideration.

The expert panel was given eight weeks to complete the initial Delphi survey. After each Delphi round, we compiled results and made additions or modifications to items based on expert panelists’ narrative suggestions. We distributed the results, including anonymized descriptions of panelist voting and comments, to the panelists along with the subsequent Delphi survey. They were given four weeks to complete each subsequent survey. We used descriptive analysis to summarize the findings from each Delphi round.

## RESULTS

**Figure 1:**
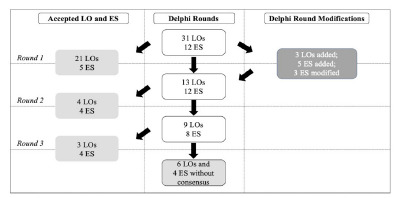
Flow diagram of the model resuscitation leadership curriculum Delphi process for learning objectives (LO) and educational strategies (ES). LO and ES meeting positive consensus (left column) were approved for addition to the final curriculum and removed from further Delphi rounds.

We recruited 29 resuscitation leadership experts to serve on the expert panel, with 12 (41.4%) agreeing to participate. Panelists represented a range of U.S. geographic regions, with the largest proportions from the Midwest and Northeast (Table 1). Panelists represented numerous training backgrounds, including critical care and other advanced fellowship or master’s-level preparation. The group also spanned a wide range of academic seniority and leadership roles. The response rate for the first Delphi round was 100% (12/12), with subsequent rounds maintaining a 92% (11/12) response rate. Results from each Delphi round are summarized in Figure 1.

After the first round, 21 learning objectives and five educational strategies met the predefined positive consensus threshold and were included in the final curriculum. Based on expert panelist feedback, three additional learning objectives and five educational strategies were introduced in subsequent Delphi rounds (Table S1). Additionally, we refined the wording of three educational strategies to better clarify instructional methodology.

In the second Delphi round, four additional learning objectives and four educational strategies reached the positive consensus threshold and were incorporated into the final curriculum (Figure 1). No further changes or additions were made for subsequent rounds. By the third and final round, three additional learning objectives and four educational strategies were accepted. A total of six learning objectives and four educational strategies did not achieve consensus and were removed from consideration.

**Figure 2:**
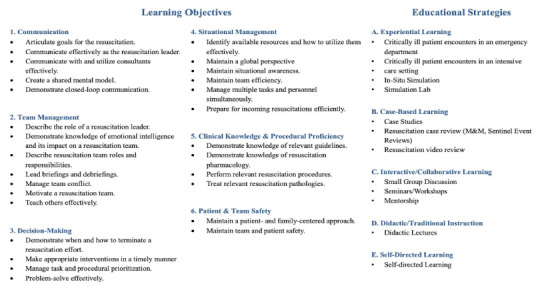
Model resuscitation leadership curriculum for emergency medicine residents.

Tables 2 and 3 present a detailed breakdown of voting for each learning objective and educational strategy across rounds. At the conclusion of the Delphi process, 28 learning objectives and 13 educational strategies were finalized for inclusion in the proposed curriculum. To support interpretation and practical application, we organized the learning objectives into six thematic domains: communication; team management; decision-making; situational management; clinical knowledge and procedural proficiency; and patient and team safety (Figure 2). We similarly grouped the educational strategies into five overarching instructional approaches: experiential learning; case-based learning; interactive and collaborative learning; didactic or traditional instruction; and self-directed learning (Figure 2).

**Figure 3:**
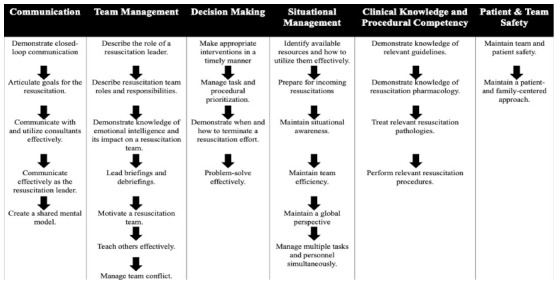
Proposed sequencing of learning objectives within the model resuscitation leadership curriculum. Sequencing was informed by Bloom’s taxonomy and shaped by author review.

The study team also developed optional supplemental resources to support programs in implementing this model curriculum, including a proposed longitudinal framework (Table 4), a rationale for the sequencing of learning objectives to progressively build knowledge and skillsets (Figure 3), and suggested educational strategies tailored to each objective type (Table S2). Progression of knowledge and skills was guided by mapping learning objectives to Bloom’s taxonomy.^40^ Learning objectives were then categorized by educational domain, and corresponding instructional strategies were selected based on the consensus-derived ideal strategy for each domain.^17^ Two external medical education experts with curriculum development experience reviewed these materials to ensure clarity, relevance, and usability. Minor revisions to Table S2 were made following expert review.

## DISCUSSION

This study represents the first structured effort to develop a model resuscitation leadership curriculum for EM residents based on expert consensus. The curriculum design is to ensure that residents are equipped to competently lead the resuscitation of critically ill patients in the ED. While previous healthcare team training programs provide generic leadership instruction, this curriculum addresses key knowledge and skills that differentiate resuscitation leadership specifically in the ED from other healthcare settings. Additionally, in contrast to previously described resuscitation leadership training approaches,^7^,^8^ this model supports the comprehensive development of resuscitation leadership knowledge and skills through a rigorous, validated, consensus-based process. By formally incorporating resuscitation leadership education into residency training, this curriculum attempts to addresses the variability, inconsistency, and potential implicit biases often associated with informal or “hidden” resuscitation leadership curricula.^1^,^14^,^41^ In doing so, it provides residents with the instruction and mentorship they both need and seek.

A central theme that emerged during the review of consensus learning objectives was the expert panel’s prioritization of competencies associated with high-performing team leadership, particularly those demonstrated to be effective in clinical environments.^16^,^42^,^43^ However, in contrast to traditional healthcare team leadership curricula, the consensus objectives also incorporated clinical and procedural resuscitation content. This distinction reflects the unique focus of a resuscitation leadership curriculum, which must integrate both leadership and clinical expertise. The curriculum’s emphasis on contextualized leadership reflects the reality of EM resuscitations where decision-making, communication, and technical execution must occur in parallel, often under conditions of uncertainty and high stakes.

Additionally, the large number of learning objectives reaching consensus highlights the multifaceted demands of resuscitation leadership and underscores the necessity of a comprehensive curricular framework. Each learning objective included in the final curriculum met a predefined and rigorous consensus threshold, reflecting the expert panel’s collective judgment that all selected competencies are essential for developing effective resuscitation leaders. This parallels the ACGME Milestones, by which EM residents are expected to achieve competence across a wide range of domains before being allowed to graduate.

Interestingly, the expert panel placed relatively little emphasis on including formal leadership theory within the curriculum. The panel may have viewed an in-depth understanding of leadership theory as non-essential for achieving competence in resuscitation leadership. This perspective aligns with existing literature suggesting that empowering leadership, characterized by shared decision-making, inclusivity, and collaborative team dynamic, is generally more effective than directive leadership styles in high-acuity healthcare settings.^9^,^44^ Several of the consensus-derived learning objectives reflect the principles of empowering leadership, including fostering a shared mental model, applying emotional intelligence, and promoting psychological safety. Although the initial literature review that informed the Delphi process included objectives related to leadership theories and traits, the expert panel ultimately determined that such theoretical content fell outside the scope of this curriculum. Future research may explore whether integrating formal leadership theoretical knowledge could enhance both the instruction and practical application of resuscitation leadership beyond the foundational competencies established during residency.

In soliciting expert input on a broad range of educational strategies, we aimed to provide programs with a flexible model from which they can select approaches best suited to their context. The panel’s selections reflected deliberate alignment between objectives and strategies. For instance, the panel did not prioritize journal clubs and podcasts due to their focus on literature updates, which the panel de-emphasized. The panel commented that educators must align educational strategies with curricular sequence and content, both of which are dynamic and program-specific. This aligns with Kern’s emphasis on iterative evaluation and continuous refinement to ensure curricular relevance.^17^ Additionally, the consensus on strategy selection reflects the panel’s collective experience as both learners and educators in resuscitation leadership, offering insights into practical application and current best practices in resuscitation leadership training.

We developed the curriculum using Kern’s six-step approach to curriculum design, a widely recognized and evidence-based framework in health professions education.^17^ This Delphi study specifically addresses the selection of learning objectives and educational strategies, corresponding to Steps 3 and 4 of Kern’s model. Implementation was intentionally not addressed (Step 5), recognizing the diversity of institutional resources, priorities, and stakeholder involvement that would influence local adaptation. To assist programs with curriculum implementation, we developed sample curriculum timelines (Table 4) and sequencing (Figure 3) to aid local implementation while allowing for personalized adaptation. We also refrained from prescribing specific evaluation and assessment strategies, recognizing the active development of resuscitation leadership assessment tools within the field. Programs may consider using entrustable professional activities to measure resuscitation leadership competency or adopt emerging simulation- and workplace-based assessment tools, depending on local faculty expertise, program priorities, and resource availability.^45^–^48^

## LIMITATIONS

There are several important limitations of this study that warrant discussion. First, the target panel size of 15–20 experts was not met, which may limit the generalizability of the resulting curriculum. Additionally, the geographic distribution of the expert panel was skewed toward the Midwest and Northeast, which may limit the broader applicability of the consensus. However, it is important to note that there is no universally accepted standard for Delphi panel size,^18^ and successful curricula in health professions education have been developed using similarly sized or even smaller expert groups.^19^–^22^ Additionally, our panelists represented a diverse range of training backgrounds and institutional affiliations enhancing the external validity and relevance of the consensus achieved. Second, alternative consensus-building methods, such as the nominal group technique, might have yielded different results as they allow for real-time, face-to-face discussion and debate on proposed items.^49^

We selected a modified Delphi process due to its methodological strengths, particularly its emphasis on anonymity, which minimizes potential biases introduced by group dynamics, professional hierarchies, or interpersonal influences. In addition, the electronic Delphi format enabled participation from a geographically varied group of experts, potentially increasing the breadth of perspectives represented.^18^ Lastly, as with all modified Delphi studies, the absence of standardized guidelines for methodology introduces potential threats to validity. These risks were mitigated by adhering closely to recently published best practices for Delphi research in health professions education.^18^ Nevertheless, the inherent limitations of the methodology, including variability in implementation and interpretation, should be acknowledged. Additionally, the literature search used to develop the initial learning objectives was limited to PubMed and did not include other databases. While not intended as a systematic review, this may have limited the breadth of source material considered in the initial objective generation process.

## Supplementary Information







## Figures and Tables

**Table 1:** Characteristics of expert panelists recruited to develop a model resuscitation leadership curriculum.

	N (%)
Region of practice	
Australia	1 (8)
United States	
Midwest	4 (33)
Northeast	5 (42)
South	1 (8)
West	1 (8)
Advanced Training	
Fellowship	
Critical Care	4 (33)
ECMO	1 (8)
Research	1 (8)
Master’s Degree	
Clinical Epidemiology	1 (8)
Public Health	1 (8)
Title	
Professor	2 (17)
Senior Staff Specialist	1 (8)
Associate Professor	4 (25)
Assistant Professor	5 (42)
Leadership Roles	
Associate Program Director	1 (8)
EM Critical Care Unit Director	2 (17)
EMS Director	1 (8)
(Assistant) Research Director	3 (25)
Resuscitation Fellowship Director	1 (8)
Survival Flight Associate Director	1 (8)
Vice Chair Academic Affairs & Faculty Development	1 (8)

*ECMO*, extracorporeal membrane oxygenation; *EM*, emergency medicine, *EMS*, emergency medical services.

**Table 2:** Delphi survey voting results for each learning objective.

Learning objective	Positive agreement (%)	Negative agreement (%)	Delphi round in which final status was determined	Final status
Describe relevant resuscitation leadership styles and when to apply them	3/11 (27.3%)	4/11 (36.4%)	3	Did not meet consensus
Articulate when to apply relevant resuscitation styles	3/11 (27.3%)	4/11 (36.4%)	3	Did not meet consensus
Describe the characteristics of an effective resuscitation leader	7/11 (63.4%)	0/11 (0%)	3	Did not meet consensus
Maintain a global perspective (open mindset to all ongoing activities)	11/12 (91.7%)	0/12 (0%)	1	Met consensus
Describe the role of a resuscitation leader	9/11 (81.8%)	0/11 (0%)	2	Met consensus
Manage multiple tasks and personnel simultaneously	11/12 (91.7%)	1/12 (8.3%)	1	Met consensus
Demonstrate knowledge of relevant guidelines (ACLS, ATLS, BLS, PALS)	11/12 (91.7%)	0/12 (0%)	1	Met consensus
Problem-solve effectively	12/12 (100%)	0/12 (0%)	1	Met consensus
Motivate a resuscitation team	10/12 (83.3%)	0/12 (0%)	1	Met consensus
Describe resuscitation team roles and responsibilities	10/12 (83.3%)	0/12 (0%)	1	Met consensus
Communicate effectively as the resuscitation leader	12/12 (100%)	0/12 (0%)	1	Met consensus
Demonstrate closed-loop communication	11/12 (91.7%)	0/12 (0%)	1	Met consensus
Communicate with and use consultants effectively	10/12 (83.3%)	0/12 (0%)	1	Met consensus
Lead briefings and debriefings	11/11 (100%)	0/11 (0%)	3	Met consensus
Maintain team efficiency	10/12 (83.3%)	0/12 (0%)	1	Met consensus
Identify available resources and how to use them effectively	10/12 (83.3%)	0/12 (0%)	1	Met consensus
Articulate goals for the resuscitation	11/12 (91.7%)	0/12 (0%)	1	Met consensus
Create a shared mental model (common understanding and internal representation of knowledge among team members)	10/12 (83.3%)	0/12 (0%)	1	Met consensus
Maintain team and patient safety	10/12 (83.3%)	0/12 (0%)	1	Met consensus
Manage team conflict	11/11 (100%)	0/12 (0%)	2	Met consensus
Perform relevant resuscitation procedures	11/12 (91.7%)	1/12 (8.3%)	1	Met consensus
Treat relevant resuscitation pathology	10/12 (83.3%)	0/12 (0%)	1	Met consensus
Have knowledge of resuscitation pharmacology	10/12 (83.3%)	1/12 (8.3%)	1	Met consensus
Maintain a patient- and family-centered approach	10/12 (83.3%)	0/12 (0%)	1	Met consensus
Maintain situational awareness	10/12 (83.3%)	0/12 (0%)	1	Met consensus
Make appropriate interventions in a timely manner	12/12 (100%)	0/12 (0%)	1	Met consensus
Understand emotional intelligence and its impact on a resuscitation team	9/11 (91.8%)	1/11 (9.1%)	3	Met consensus
Know when and how to terminate a resuscitation effort	10/12 (83.3%)	0/12 (0%)	1	Met consensus
Able to efficiently prepare for incoming resuscitations	11/11 (100%)	0/11 (0%)	2	Met consensus
Have knowledge of literature on the impact of resuscitation leadership on patient outcomes	2/11 (18.2%)	3/11 (27.3%)	3	Did not meet consensus
Effectively teach others	11/11 (100%)	0/11 (0%)	3	Met consensus
Learning objective	Positive agreement (%)	Negative agreement (%)	Delphi round in which final status was determined	Final status
*Articulate one’s preferred resuscitation leadership style	4/11 (36.4%)	3/11 (27.3%)	3	Did not meet consensus
*Understand the impact of the resuscitation-space physical environment on a resuscitation	8/11 (72.7%)	1/11 (9.1%)	3	Did not meet consensus
*Manage task and procedural prioritization	11/11 (100%)	0/11 (0%)	2	Met consensus

* Item added in second Delphi survey.

**Table 3:** Delphi survey voting results for each educational strategy.

Educational strategy	Positive agreement (%)	Negative agreement (%)	Delphi round in which final status was determined	Final status
Didactic lectures	9/11 (81.8%)	0/11 (0%)	2	Met consensus
Small-group discussions	12/12 (100%)	0/12 (0%)	1	Met consensus
Seminars/workshops	10/12 (83.3%)	0/12 (0%)	1	Met consensus
Case studies	11/12 (91.7%)	0/12 (0%)	1	Met consensus
Self-directed learning	10/11 (90.9%)	0/11 (0%)	3	Met consensus
On-shift education in the ED	12/12 (100%)	0/12 (0%)	1	Met consensus
Mentorship	10/12 (83.3%)	0/12 (0%)	1	Met consensus
Journal Club	7/11 (63.6%)	1/11 (9.1%)	3	Did not meet consensus
Critically ill patient encounters in an intensive care setting (formerly Critical Care rotation)	10/11 (90.9%)	0/11 (0%)	3	Wording modified, met Consensus
Critically ill patient encounters in a prehospital setting (formerly EMS rotation)	7/11 (63.6%)	2/11 (18.2%)	3	Wording modified, did not meet Consensus
Critically ill patient encounters in an emergency department setting (formerly Resuscitation rotation)	10/11 (90.9%)	0/11 (0%)	2	Wording modified, met consensus
Reading (textbooks, journals, etc)	7/11 (63.6%)	0/11 (0%)	3	Did not meet consensus
*Simulation lab	10/11 (90.9%)	1/11 (9.1%)	3	Met consensus
*In-situ simulation	9/11 (81.8%)	1/11 (9.1%)	3	Met consensus
*Resuscitation video review	9/11 (81.8%)	0/11 (0%)	2	Met consensus
*Resuscitation case review (M&M, sentinel event reviews)	10/11 (90.9%)	0/11 (0%)	2	Met consensus
*Podcasts/high-quality blogs	6/11 (54.5%)	2/11 (18.2%)	3	Did not meet consensus

* Item added in second Delphi survey.

*ED*, emergency department; *EMS*, emergency medical services; *M&M*, morbidity and mortality.

**Table 4:** Three-phase implementation framework of the model resuscitation leadership curriculum.

	Phase 1	Phase 2	Phase 3
Communication	Demonstrate closed-loop communication.? Articulate goals for the resuscitation.	Communicate with and use consultants effectively. Communicate effectively as the resuscitation leader.	Create a shared mental model.
Team management	Describe the role of a resuscitation leader. Describe resuscitation team roles and responsibilities. Demonstrate knowledge of emotional intelligence and its impact on a resuscitation team.	Motivate a resuscitation team. Lead briefings and debriefings.	Manage team conflict. Teach others effectively.
Decision-making	Make appropriate interventions in a timely manner	Demonstrate when and how to terminate a resuscitation effort.	Problem-solve effectively.
Situational management	Identify available resources and how to use them effectively.	Efficiently prepare for incoming resuscitations Maintain situational awareness. Maintain team efficiency.	Maintain a global perspective Manage multiple tasks and personnel simultaneously.
Clinical knowledge and procedural competency	Demonstrate knowledge of relevant guidelines. Demonstrate knowledge of resuscitation pharmacology.	Treat relevant resuscitation pathologies. Perform relevant resuscitation procedures.	
Patient and team safety	Maintain team and patient safety.	Maintain a patient- and family-centered approach.	
