# Supplementary material for: Model Resuscitation Leadership Curriculum for Emergency Medicine Residents: Modified Delphi Study
Source: West J Emerg Med. 2026 Mar 1;27(2):402–12. doi: 10.5811/westjem.50811 (PMC13016047; doi:10.5811/westjem.50811)
Supplement: Supplementary file 1 [file wjem-27-402-s001.pdf]

Table S1. Delphi panel comments by Delphi round

Delphi Round	Survey	Comment
1	Learning Objective	I think it is important to have awareness of your own resuscitation style. It is not necessary to assume a "classic" resuscitation style- but to find which is most aligned with your personality so it is effective and does not encourage feelings of "imposter syndrome".
1	Learning Objective	Ability to remain hands off the patient, maintain team awareness and not pulled into task specific situations
1	Learning Objective	More specifically regarding debriefs, knowing and utilizing a cold debrief vs a warm debrief. Also, knowing how to convey bad news after a poor resuscitation outcome to a family is critical skill. Overall, communication with family members is important for resuscitation leader.
1	Learning Objective	Understand effect of physical environment on resuscitation and the ways it can be optimized for resuscitation effectiveness
1	Learning Objective	Assume #13 (Able to communicate with and utilize consultants effectively) is meant to manage team members in addition to consultants, and that these are different. Don't see where team members are assigned and that leader assures that key roles are filled. Is this #10 (Describe resuscitation team roles and responsibilities) #26 (Make appropriate interventions in a timely manner), #22 (Able to treat relevant resuscitation pathology) Question i have is whether this is meant to assure that appropriate interventions are performed in a timely manner, rather than the team leader does them (e.g. thoracotomy, cric) Don't see anything about prioritizing tasks and procedures (e.g ortho splints legs while airway is being controlled, nurses place 5 IVs rather than allowing team member space to place chest tube, etc).
1	Learning Objective	Proactively solicits feedback on leader performance Able to self-reflect. Develops metacognitive skills, including being mindful of switching between system 1 and 2 thinking
1	Learning Objective	can balance workload within team can create contingency plans can set priorities for taskwork monitors the team's progress toward goals
1	Educational Strategies	Simulation training (if not included under the seminars/workshops category)
1	Educational Strategies	resuscitation and post-code debrief which may be helpful as you create your curriculum. I would also add, self-reflection as a different method. Understanding how you lead, garnering feedback from team members and incorporating constructive comments can yield great results.
1	Educational Strategies	Advanced team dynamics training (like TeamStepps), in-situ resuscitation simulation scenarios with multidisciplinary teams
1	Educational Strategies	Podcasts and high quality blogs with careful interpretation
1	Educational Strategies	Simulation sessions.
1	Educational Strategies	Simulation
1	Educational Strategies	#44 (Reading) hard to argue with reading, but assume you mean that there's a core curriculum for this topic. #41 and #43 (critical care & resuscitation rotations). What's the difference here? Not sure what incremental benefit of #35-43 (educational strategy options) are for residency in general and specific to leadership curriculum. Also need critical evaluation of leadership role (tried to do this already I know, but probably need for team leader activities, rather than routine cases).
1	Educational Strategies	simulation video-review
1	Educational Strategies	the inclusion of the rotations (CC, EMS) don't make sense here - it is dependent upon the content. That is true for almost all items so I'm not

		sure what you hope to gain here. I also don't know what a resuscitation rotation entails. I am unclear why you don't include simulation-based training. That is a large category with multiple subcategories that might be worth exploring.
2	Learning Objectives	Communication ability while under duress and with limited cognitive bandwidth
2	Learning Objectives	Metacognition, Zero Point Survey
2	Learning Objectives	I think this may be covered previously, but handling other disciplines/consultants during resuscitation is critical
2	Educational Strategies	Video would be great if available
2	Educational Strategies	Mode of learning depends on content and how learning is scaffolded. This question in my opinion does not help you address the issue of how to teach resuscitation leadership, it is way too simplistic
