# Supplementary material for: Model Resuscitation Leadership Curriculum for Emergency Medicine Residents: Modified Delphi Study
Source: West J Emerg Med. 2026 Mar 1;27(2):402–12. doi: 10.5811/westjem.50811 (PMC13016047; doi:10.5811/westjem.50811)
Supplement: Supplementary file 3 [file wjem-27-402-s003.pdf]

Table S3. Categorization of learning objectives and corresponding ideal educational strategies by objective domain

	Cognitive Domain	Psychomotor Domain	Affective Domain
**Communication**	Articulate goals for the resuscitation.<br><br>Create a shared mental model.	Demonstrate closed-loop communication<br><br>Communicate with and utilize consultants effectively.<br><br>Communicate effectively as the resuscitation leader.	
**Team Management**	Describe the role of a resuscitation leader.<br><br>Describe resuscitation team roles and responsibilities.<br><br>Demonstrate knowledge of emotional intelligence and its impact on a resuscitation team.	Lead briefings and debriefings.<br><br>Manage team conflict.<br><br>Teach others effectively.	Motivate a resuscitation team.
**Decision Making**	Problem-solve effectively.	Demonstrate when and how to terminate a resuscitation effort.<br><br>Make appropriate interventions in a timely manner<br><br>Manage task and procedural prioritization.	
**Situational Management**	Identify available resources and how to utilize them effectively.	Efficiently prepare for incoming resuscitations<br><br>Maintain situational awareness.<br><br>Maintain team efficiency.<br>Maintain a global perspective<br><br>Manage multiple tasks and personnel simultaneously.	
**Clinical Knowledge & Procedural Competency**	Demonstrate knowledge of relevant guidelines.<br><br>Demonstrate knowledge of resuscitation pharmacology.	Treat relevant resuscitation pathologies.<br><br>Perform relevant resuscitation procedures.	
**Patient & Team Safety**			Maintain a patient- and family-centered approach.

			Maintain team and patient safety.
**Suggested Educational Strategy**	Didactic/Traditional<br><br>Instruction, Self-Directed Learning<br><br>Interactive/Collaborative Learning	Experiential Learning<br><br>Case-Based Learning<br><br>Interactive/Collaborative Learning	Experiential Learning<br><br>Case-Based Learning,<br><br>Interactive/Collaborative Learning
